# Band‐Selective, Low‐Dispersion Terahertz Attenuators Based on 3D Bulk Metamaterials Leveraging De‐latticed Cross‐Bar Resonator Ensembles

**DOI:** 10.1002/nap2.70203

**Published:** 2026-08-13

**Authors:** Zhen Liu, Chikaho Nagashima, Yoshiaki Kanamori

**Affiliations:** ^1^ Graduate School of Engineering Tohoku University Sendai Japan

**Keywords:** 3D metamaterial, bulk composite, cross‐bar resonator, isotropic, terahertz attenuator

## Abstract

Terahertz (THz) optical components for power attenuation and dispersion management require materials that provide isotropic and band‐selective control of electromagnetic waves. However, two‐dimensional (2D) metasurfaces based on coherent lattice coupling exhibit polarization anisotropy and angular dispersion, which lead to phase distortion. Here, three‐dimensional (3D) bulk metamaterials (MMs) functioning as THz attenuators are proposed and experimentally demonstrated based on a de‐latticing strategy inspired by amorphous natural materials. Periodic arrays of cross‐bar meta‐atoms are encapsulated by cyclo‐olefin polymer (COP) as randomly oriented cubic meta‐grains and dispersed within a COP matrix, forming an amorphous composite that eliminates in‐plane coherence. The fabricated samples exhibit band‐selective and thickness‐tunable attenuation with a smooth refractive‐index variation across 0.3–0.4 THz. Compared with the 2D metasurface, the dispersion slope Δn/Δf decreases from 8.37 to 0.36 at the resonant frequency, indicating an order‐of‐magnitude suppression of phase dispersion. The attenuation scales continuously with thickness owing to incoherent resonant scattering from randomly oriented dipoles, providing a practical pathway to thickness‐dependent and band‐selective response. This work demonstrates an isotropic, low‐dispersion, and thickness‐tunable 3D bulk MMs THz attenuator that overcomes the polarization anisotropy and strong dispersion of conventional 2D attenuators, offering a practical pathway toward integrated THz photonic systems.

## Graphical abstract

This paper presents 3D bulk metamaterials in which diced cross‐bar meta‐grains are randomly dispersed in a polymer matrix by molding. The de‐latticed composite provides isotropic and band‐selective THz attenuation with over tenfold dispersion suppression compared to the periodic metasurface. The attenuation depth is continuously tunable by sample thickness, enabling alignment‐free operation for practical THz photonic systems.

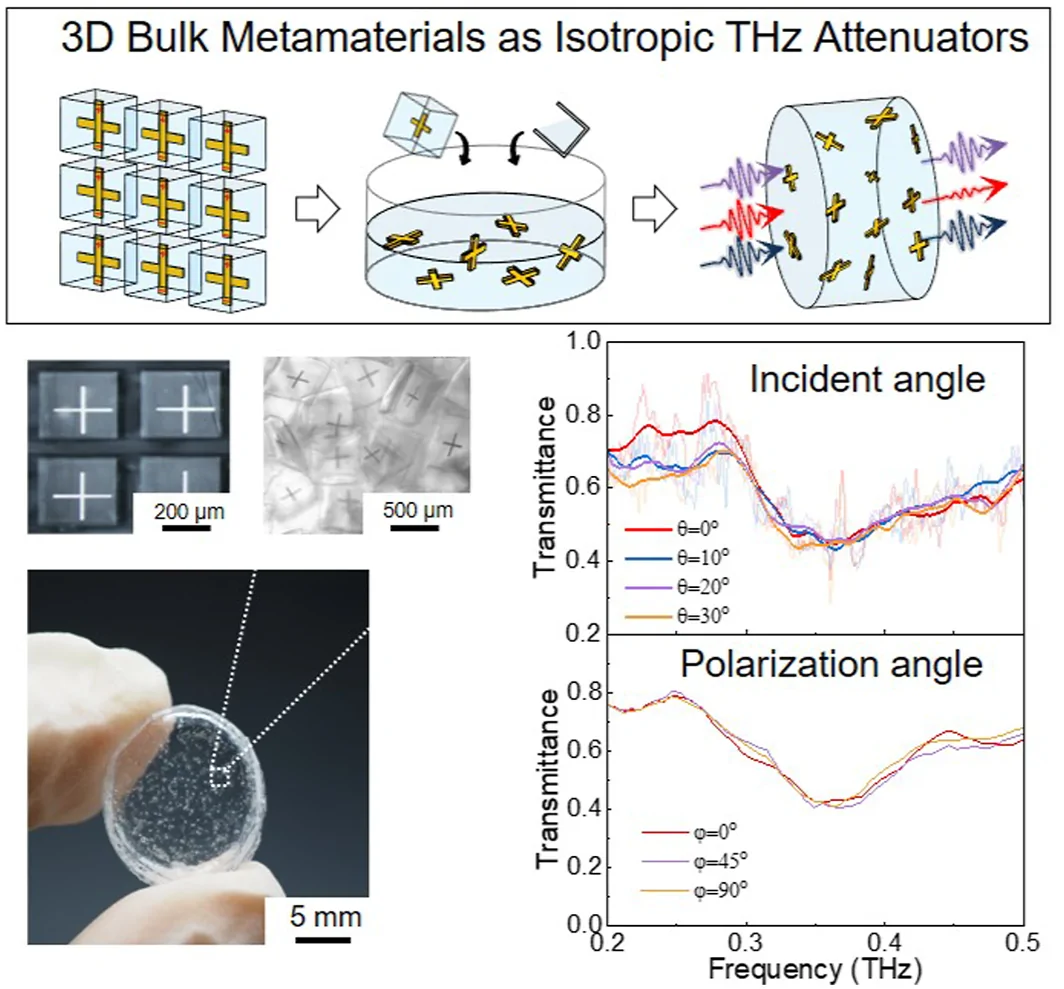

## 1 Introduction

Terahertz (THz) waves have attracted significant attention in recent decades for applications in nondestructive inspection, sensing, and imaging across diverse industrial and scientific sectors [1, 2, 3]. Moreover, the 0.2–0.5 THz range serves as a key frequency band for next‐generation devices with the advent of sixth‐generation (6G) wireless communications [4]. Implementing such systems requires optical components capable of precisely manipulating electromagnetic waves, such as power attenuators, dispersion modulators, and polarizers. However, most natural materials interact weakly with THz radiation and therefore provide limited control over their transmittance and refractive index [5, 6, 7, 8].

Metamaterials (MMs), composed of periodic arrays of subwavelength meta‐atoms, have been developed as artificial media with engineered electromagnetic responses. Their optical characteristics can be tailored by controlling the geometry and orientation of the constituent meta‐atoms, enabling spectral modulation that is unattainable in natural materials [9, 10, 11, 12, 13]. A variety of meta‐atom geometries have been explored, including electric‐dipole elements such as cross‐bars and cut wires, magnetic resonators based on split‐ring resonators (SRRs), and complementary apertures patterned in metallic films [14, 15, 16, 17, 18]. These engineered structures exhibit permittivity and permeability ranging from near‐zero to ultrahigh refractive index, which are determined by the local resonance and coupling strength [19, 20, 21]. The dynamic control of resonance amplitude and frequency can be further realized by combining active materials such as graphene, phase‐change materials, and microelectromechanical systems (MEMS) [22, 23, 24, 25, 26, 27]. These developments have established MMs as a flexible platform for electromagnetic wave manipulation, yielding band‐selective absorbers, spectral filters, polarization rotators, and emission‐enhancing elements [28, 29, 30, 31, 32, 33].

Despite these advances, the periodic meta‐atoms in two‐dimensional (2D) metasurfaces induce polarization anisotropy and angular dispersion through near‐field coupling and lattice coherence [34, 35, 36]. Moreover, the confined light–matter interaction within a thin layer restricts the optical path length, thereby reducing the attainable attenuation and modulation depth. Recent research has focused on extending MMs design into three dimensions (3D) to overcome these limitations of planar architectures. Approaches such as multilayer stacking, self‐aligned membrane projection lithography, and focused ion‐beam milling have enabled out‐of‐plane architectures and increased structural thickness [37, 38, 39, 40]. However, the limited throughput of such techniques poses challenges for scalable fabrication and industrial applications. We have previously reported a scalable alternative approach in which metallic SRRs are randomly dispersed within a cyclo‐olefin polymer (COP) matrix [41]. The random orientation statistically averages the meta‐atom response, yielding isotropic optical behavior without alignment constraints. In addition to isotropy, 3D MMs provide greater design freedom in thickness and volume, enabling longer interaction lengths between THz waves and resonant elements [42]. These earlier works used the random‐dispersion platform to engineer a large dispersion, whereas here the de‐latticing strategy is used to suppress it, so that the bulk operates as a low‐dispersion attenuator. This volumetric degree of freedom enables controllable attenuation with low dispersion through the cumulative modulation of amplitude and phase along the propagation path. For instance, conventional absorptive or interference‐based attenuators remain narrowband and polarization‐sensitive, whereas resonant MM absorbers often introduce strong dispersion [43, 44, 45]. A single random layer lowers the dispersion but is too thin to attenuate strongly, so reaching both strong attenuation and low dispersion requires extending the de‐latticed medium into 3D. Furthermore, although cascading or stacking multiple planar metasurfaces can broaden the attenuation band and manage dispersion, such multilayer structures remain fundamentally anisotropic and sensitive to the incidence angle [46, 47, 48].

In this study, we propose and experimentally demonstrate de‐latticed 3D bulk MMs composed of randomly oriented cross‐bar resonators dispersed in a COP matrix, as illustrated in Figure 1a. In 2D metasurfaces, the optical response arises from the localized dipole resonance (DR) of each cross‐bar and the surface‐lattice resonance (SLR) associated with in‐plane diffraction, where the SLR strongly depends on the polarization angle θ and incidence angle ϕ, as shown in Figure 1b,c, respectively. Inspired by the amorphous structure of isotropic natural materials, we proposed a de‐latticing strategy to move beyond the limitation of 2D metasurfaces. The metasurface was diced into individual discrete meta‐grains and randomly dispersed within a COP matrix (Figure 1d). This approach eliminates in‐plane periodicity and suppresses coherent coupling between neighboring dipoles while leaving the intrinsic dipole resonance intact. The cross‐bar is adopted because its dipole resonance is simple and free of higher‐order modes in the operating band, which allows the de‐latticing strategy to be demonstrated clearly. The ensemble‐averaged response thus becomes polarization independent, governed by incoherent dipole scattering. The fabricated 3D bulk MMs exhibit broadened and thickness‐dependent attenuation across 0.3–0.4 THz. Compared with the 2D metasurface, the 3D bulk MMs show a more than tenfold smaller dispersion slope, defined as Δn/Δf, while maintaining a clear thickness‐dependent attenuation band. This indicates that the proposed de‐latticing strategy effectively suppresses phase dispersion while preserving the tunability of the dipole resonance. The proposed isotropic and scalable 3D bulk MMs are expected to provide band‐selective, polarization‐insensitive, and low‐dispersion THz wave attenuation, offering a practical route toward integration in next‐generation THz photonic systems.

**FIGURE 1:**
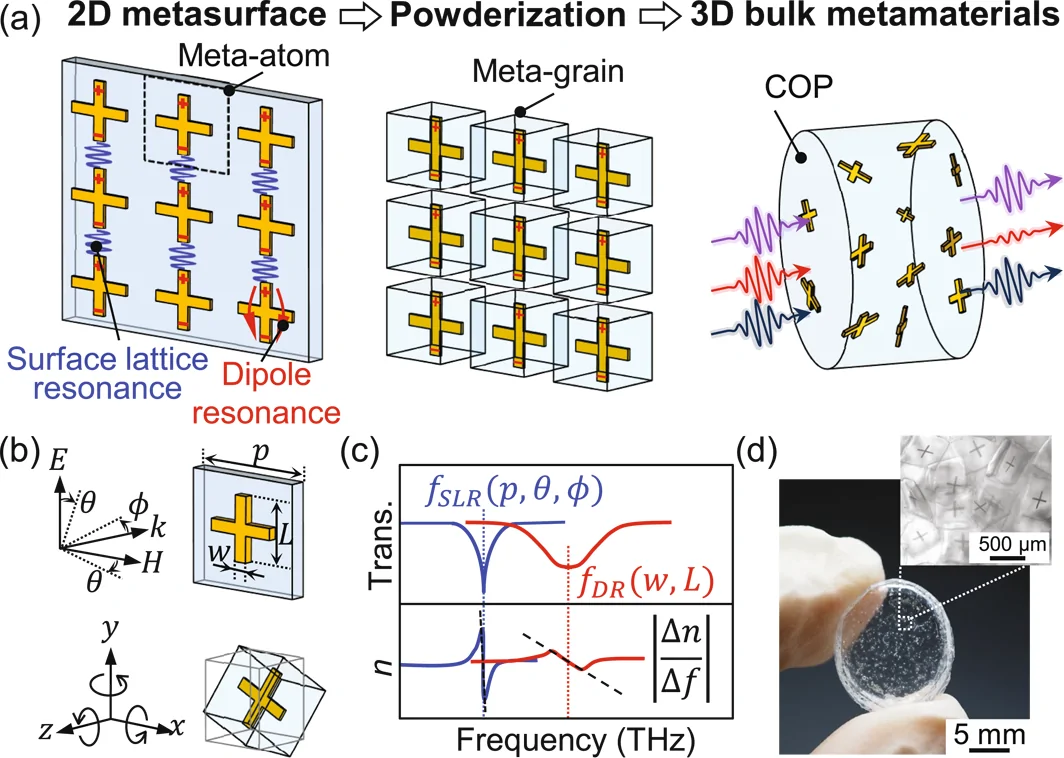
(a) Concept of the proposed 3D bulk MMs in which the 2D metasurface is powderized into individual meta‐grains based on the unit cell of meta‐atom and randomly dispersed in a COP matrix to form isotropic 3D bulk MMs functioning as a THz attenuator. (b) Schematic of the geometry and orientation of an individual cross‐bar resonator. (c) Illustration of the spectral features and refractive‐index dispersion of the SLR and DR. The dispersion slope is defined as the variation of the refractive index Δn with respect to the resonant frequency bandwidth Δf. (d) Photograph of the fabricated 3D bulk MMs. The inset shows a magnified microscopic image revealing random distributed cross‐bar embedded in the COP matrix.

## 2 Design and Numerical Analysis

To model the resonance behavior of the proposed 3D bulk MMs composed of cross‐bar resonators, numerical simulations were performed using CST Studio Suite (Dassault Systèmes S.E.). The simulations were conducted for a periodic 2D metasurface consisting of cross‐bar unit cells embedded in a COP medium. The frequency–angle dependence of the transmittance and the corresponding effective refractive index of the periodic array were extracted from the calculated S‐parameters. The results are shown in Figure 2a,b, respectively. The incident wave vector *k* was oriented normal to one arm of the cross, and the incidence angle ϕ was varied within the incidence plane, as illustrated in the inset of Figure 2a. The geometric parameters were set to p=451μm, L=260μm, w=25μm, and t=200nm, where p, L, w, and t denote the lattice period, arm length, arm width, and metal thickness, respectively. Aluminum (Al) was chosen as the metallic component with an electrical conductivity of $3.56\times 10^{7}\;S\;m^{-1}$. The refractive indices of the surrounding media were defined as $n_{\mathrm{matrix}}=1.52$ for COP. The dicing pitch p was set larger than the resonator length L, so that the metallic cross‐bar was fully embedded in the COP cube and adjacent meta‐grains were separated by the dielectric. This geometry suppresses near‐field coupling between neighboring grains, and the optical response is therefore governed mainly by the localized resonance of each embedded cross‐bar resonator.

**FIGURE 2:**
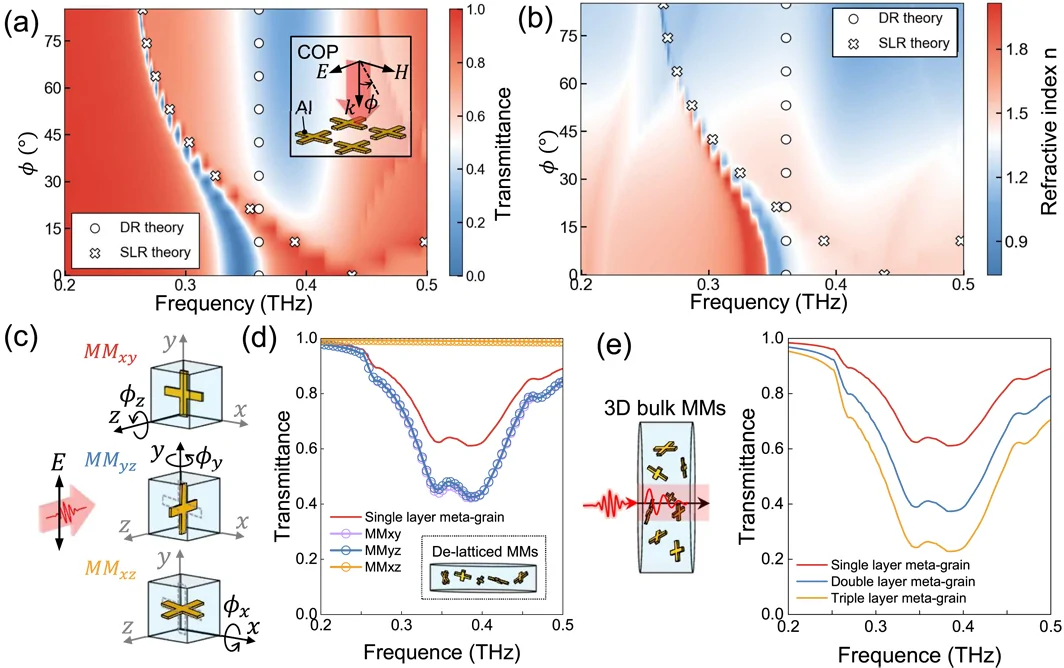
(a) Simulated angular–frequency dispersion map of the transmittance for periodic arrays of cross‐bar resonators embedded in a COP medium. (b) Retrieved effective refractive index n corresponding to the result in (a). (c) Schematic illustration of three orientations of the meta‐grain. (d) Calculated transmittance spectra for MM_xy, MM_yz, MM_xz, and the single‐layer average of the de‐latticed configuration shown in the inset. (e) Calculated transmittance spectra for different effective layer numbers N, obtained using Equation (4).

As shown in Figure 2a,b, the calculated angular dispersion exhibits two dominant resonance branches. The first corresponds to the DR arising from the fundamental current oscillation along the finite metallic arms. Its frequency can be approximated by a Fabry–Pérot‐type half‐wave model,

(1) $$f_{\text{DR}}=\frac{c}{2\;n_{\text{eff}}\;L_{\text{eff}}},\;L_{\text{eff}}=L+2\Delta ,\;\Delta =\eta w$$

where c denotes the speed of light, $n_{\text{eff}}$ is the local effective refractive index, and Δ represents the end‐fringe correction that accounts for the capacitive field extension at the dipole ends. For estimation, $n_{\text{eff}}=1.52$ was used to represent the material matrix of COP, and an empirical correction factor η=0.3 was applied to account for finite‐end effects. This resonance is highly localized and therefore exhibits negligible angular dependence, as indicated by the nearly vertical circles. In addition to this localized mode, the periodic array of meta‐atoms gives rise to a collective SLR originating from in‐plane diffraction. It is governed by in‐plane momentum conservation and depends on the lattice period, incident angle, and the refractive indices of the surrounding media:

(2) $$f_{\text{SLR}}^{\left(\pm m\right)}\left(\phi \right)=\frac{c}{p}\;\frac{m}{n_{\mathrm{matrix}}\pm \sin\;\phi },$$

where m is the diffraction order, and the ± signs denote opposite in‐plane diffraction directions. The first‐order branches (m=1) are marked by cross symbols.

Strong coupling occurs when the DR and SLR frequencies overlap, as evidenced by the hybridization observed at ϕ=20° in Figure 2a. This interaction leads to an avoided crossing and a broad suppression of transmission around 0.33–0.36 THz at ϕ=20°. Figure 2b shows the retrieved effective refractive index $n_{\text{eff}}\left(\phi ,f\right)$, which varies markedly within the hybridization band around 0.33–0.36 THz when ϕ<20°, reflecting rapid phase accumulation caused by coupling between the localized and diffractive modes. The correlated behavior of T(ϕ,f) and $n_{\text{eff}}\left(\phi ,f\right)$ confirms that the observed dispersion arises from the interplay between these two resonant pathways. When this long‐range periodicity is removed in the de‐latticed ensemble of randomly dispersed cross‐bar resonators, the lattice‐mediated coupling is statistically averaged out, whereas the intrinsic dipole resonance is preserved. The optical response then becomes broader and smoother, with markedly reduced dispersion, which forms the physical basis for the 3D bulk MMs. Quantitatively, removing the lattice lowers the transmission‐phase slope at resonance from about 650 to below 50 rad/THz, linking the suppression of the SLR to the reduced dispersion; the full coupled‐dipole analysis is given in Supporting Information S1: Sections S1–S3.

The 3D isotropic response follows from orientational averaging within effective medium theory [49, 50]: since each cross‐bar resonator is subwavelength and acts as a localized electric dipole, the macroscopic effective polarizability of the random ensemble equals one‐third of the trace of the individual polarizability tensor. We therefore simulated three orthogonal orientations (${\text{MM}}_{\text{xy}}$, ${\text{MM}}_{\text{yz}}$, ${\text{MM}}_{\text{xz}}$) by aligning the incident *k*‐vector and polarization with the principal axes of the cross‐bar (Figure 2c). For each orientation i∈{xy,yz,xz}, the angle‐averaged transmittance is

(3) $$\langle T_{i}{\left(f\right)\rangle }_{\phi }={\langle |S_{21,i}\left(\phi ,f\right){|}^{2}\rangle }_{\phi },$$

and the bulk transmittance is then

(4) $$T_{\text{bulk}}\left(f\right)=\sum_{i}\beta_{i}{\langle T_{i}\left(f\right)\rangle }_{\phi },$$

where $\beta_{\text{xy}}=8/12$, $\beta_{\text{yz}}=2/12$, and $\beta_{\text{xz}}=2/12$. A cubic meta‐grain has 12 independent configurations; the $C_{4v}$ symmetry of the cross‐bar makes 8 of them equivalent to ${\text{MM}}_{\text{xy}}$, with the remaining 4 split equally between ${\text{MM}}_{\text{yz}}$ and ${\text{MM}}_{\text{xz}}$. The full derivation is given in Supporting Information S1: Section S2.

Figure 2d shows the transmittance obtained from the numerical model. ${\text{MM}}_{\text{xy}}$ and ${\text{MM}}_{\text{yz}}$ exhibit clear resonance peaks because the incident electric field drives dipole currents along the metallic arms in those planes. In contrast, ${\text{MM}}_{\text{xz}}$ shows little response as the field is nearly orthogonal to the resonator arms. A Savitzky–Golay filter was applied to the spectra in Figure 2d,e to show their continuous trends after angular averaging. Based on Equation (4), the transmittance of an incoherent de‐latticed MMs with an effective thickness equivalent to a single meta‐grain layer can be expressed as $T_{\text{bulk}}\left(f\right)$. Given that the thickness of each meta‐grain exceeds the propagation length at the resonance frequency, the total transmittance of the 3D bulk MMs composed of N such layers can be approximated as

(5) $$T_{\text{bulk}}^{\left(N\right)}\left(f\right)={\left[\;T_{\text{bulk}}\left(f\right)\;\right]}^{N},$$

where N≈t/p denotes the number of meta‐grain layers corresponding to the sample thickness t and the characteristic grain size p.

The transmission spectra of the 3D bulk MMs with various effective meta‐grain layers are shown in Figure 2e. These results confirm that orientation averaging over randomly distributed resonators effectively produces an isotropic optical response. Moreover, tunable transmission attenuation can be achieved by controlling the number of effective meta‐grain layers. The de‐latticed numerical model computes the orientation‐averaged transmittance (Equations (3), (4), (5)); no effective refractive index is extracted from this procedure. The effective refractive index reported in Section 4 is obtained from THz‐TDS measurements of the fabricated bulk samples.

## 3 Formation of 3D Bulk MMs

3D bulk MMs composed of cross‐bar meta‐grains were fabricated following the process illustrated in Figure 3a. First, a 188 μm‐thick COP film (Zeonor Film ZF14, Nippon Zeon Co. Ltd.) for the first layer was hydrophilized by UV illumination using a UV‐ozone cleaner (UV Ozone Cleaner NL‐UV253, LaserTechno Corporation) (Figure 3a,i). The UV irradiation time was set to 30 min. Next, double‐sided adhesive tape (300LSE 9495LE, 3M Corporation), UV‐peelable dicing tape (Adwill D‐218, LINTEC Corporation), and the hydrophilized COP film were sequentially attached onto a 4‐inch dummy silicon wafer (Figure 3a,ii). A 200‐nm thick Al layer was then deposited on the surface via sputtering (Figure 3a,iii), and cross‐bar meta‐atom patterns were defined by photolithography and wet etching (Figure 3a,iv). Subsequently, a 188 μm thick COP film coated with a 75 μm thick adhesive layer (HSC563, NEW TAC KASEI Co. Ltd.) was laminated on top of the patterned Al structures (Figure 3a,v). After UV irradiation, the UV‐peelable dicing tape and the underlying layers were removed, yielding a metasurface sheet in which the Al cross‐bar meta‐atoms were encapsulated between two COP resin layers. The obtained metasurface sheet was fixed on a dicing tape and diced into individual meta‐grains using a diamond blade (thickness: 100 μm, R07‐SD500‐BB200‐75 52×0.1A2×40, DISCO Corporation) (Figure 3a,vi).

**FIGURE 3:**
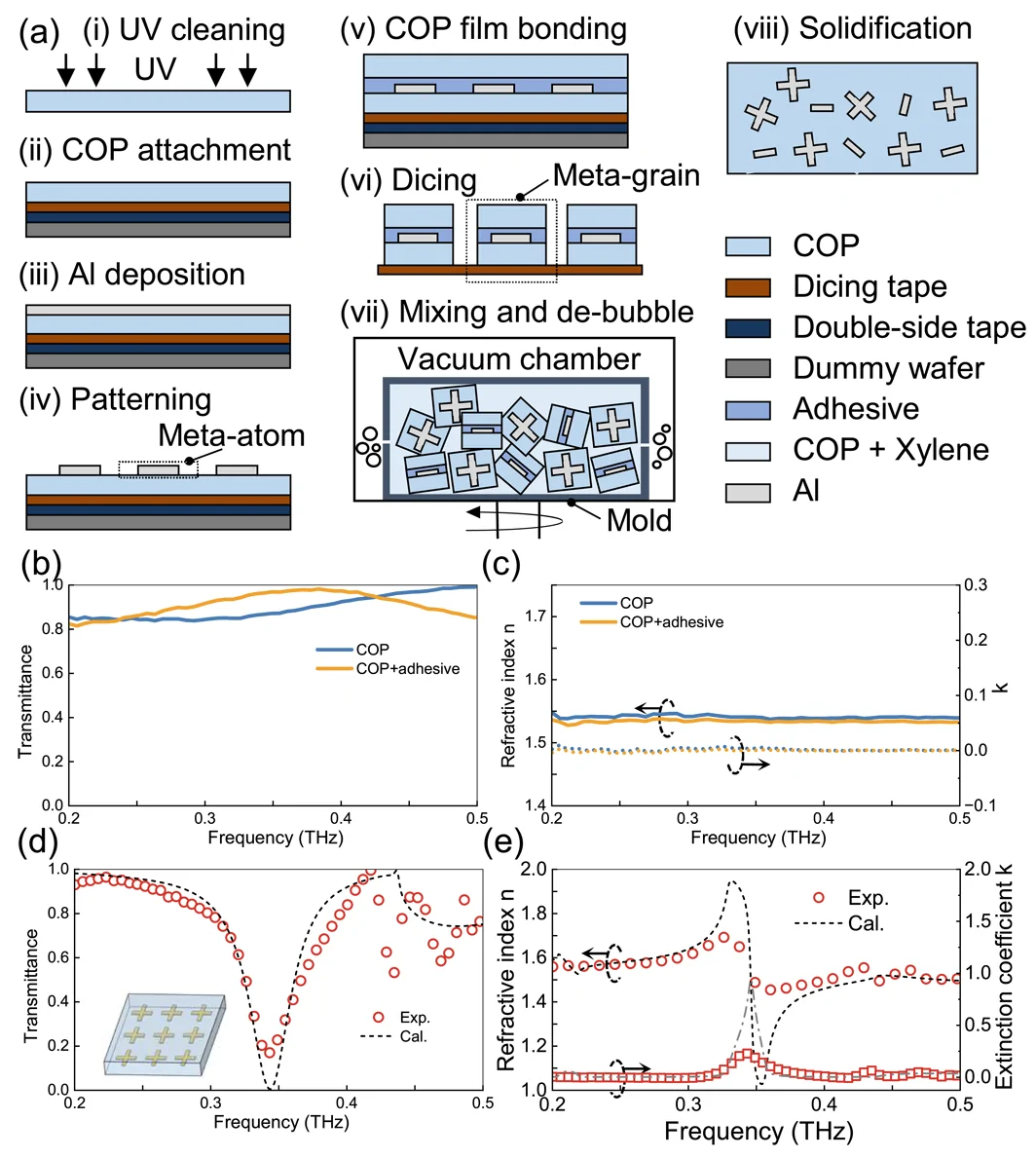
(a) Fabrication process of the 3D bulk MMs. (b, c) THz‐TDS measured transmittance and dispersion characteristics of the COP and COP/adhesive layers, respectively. (d, e) Measured and simulated transmission and dispersion characteristics of the undiced 2D periodic cross‐bar metasurface, as illustrated in the inset.

For bulk formation, 0.30 g of the meta‐grains was placed into a cylindrical metal mold (inner diameter: 13 mm) and mixed with a 20 wt% solution of COP particles (ZEONEX K22R, Nippon Zeon Co. Ltd.) in xylene, as shown in (Figure 3a,vii). The mixture was subjected to vacuum stirring and degassing using a rotation–revolution mixer (ARV‐310, THINKY Corporation), followed by drying and solidification under ambient conditions for 24 h (Figure 3a,viii). The relatively high viscosity of the COP solution suppresses aggregation and sedimentation during this process, helping to retain a uniform random dispersion of the meta‐grains throughout the bulk samples. A 3D bulk MMs sample was thus obtained after demolding.

The transmission and dispersion of the COP layers were characterized using terahertz time‐domain spectroscopy (THz‐TDS) (TeraProspector, Nippo Precision Co. Ltd.). The optical constants were retrieved from the complex transmission using the total film thicknesses (188 μm for bare COP and 263 μm for the COP/adhesive laminate). The 75 μm adhesive layer is transparent in this frequency range and its refractive index is close to that of COP (n≈1.52), so the effective optical constants of the laminate remain nearly unchanged from those of bare COP. During data processing, the full time‐domain waveform was retained without time‐gating to preserve frequency resolution. As a result, slight Fabry–Pérot fringes appear in the frequency‐domain transmittance spectra (Figure 3b), arising from internal reflections within the parallel dielectric layers. These fringes do not affect the extracted optical constants or the main attenuation features of the structure. The transmission and dispersion characteristics of the undiced 2D periodic cross‐bar metasurface were measured and are shown in Figure 3d,e. In the simulation, a cross‐bar sandwiched between COP layers was modeled as a periodic unit cell with the fabricated in‐plane lattice period of 551μm, whereas the unit cell thickness was equal to the meta‐grain dimension p, ensuring a cubic geometry for each meta‐grain. The experimental spectra have a good agreement with the simulated resonant frequency and dispersion trend, confirming that the fabrication process accurately preserved the designed geometry. For the fabricated metasurface, the first‐order SLR at normal incidence lies near 0.36 THz and nearly overlaps with the DR, so the two resonances merge into a single broadened dip. The additional transmission drop above ∼0.44 THz is attributed to the finite divergence of the focused THz beam in the experiment. Collectively, these results indicate both the optical quality of the COP‐based substrate and the precise dimensional control achieved in the fabrication.

The optical images of the overall fabrication sequence of the bulk MMs are illustrated in Figure 4. The process can be conceptually divided into three stages: (*i*) structurization, in which the designed meta‐atom patterns are transferred onto the COP substrate; (*ii*) powderization, where the structured film is diced into micron‐scale meta‐grains; and (*iii*) solidification, in which the dispersed meta‐grains are randomly embedded within a COP matrix and re‐solidified into an optical component. This sequence transforms a 2D metasurface into isotropic 3D bulk MMs while maintaining the intrinsic resonant characteristics of the design.

**FIGURE 4:**
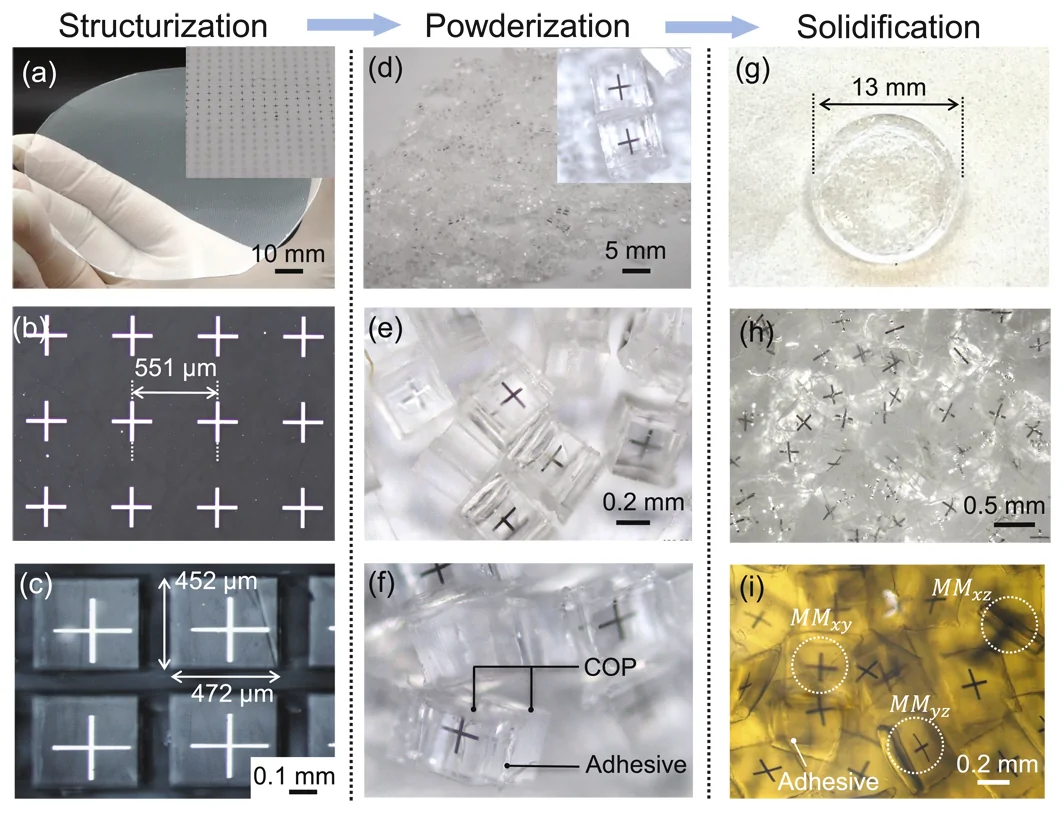
Structurization, powderization, and solidification processes for fabricating the 3D bulk MMs. (a–c) Lithographic formation of cross‐bar meta‐atom arrays on a COP substrate. (d–f) Dicing of the metasurface into COP meta‐grain microcubes, each containing an Al cross‐bar sandwiched between two COP layers with an adhesive interlayer. (g–i) Reconstitution of the diced meta‐grains into bulk MMs.

In the structurization stage, an array of Al cross‐bar resonators was lithographically patterned on a COP substrate to form a metasurface sheet (Figure 4a–c). The metasurface exhibited a lattice pitch of 551μm (Figure 4b). After dicing, the lateral dimensions of the COP microcubes were 462μm×472μm×451μm (Figure 4c), consistent with the design values and confirming accurate transfer of the in‐plane geometry to the cubic meta‐grains.

In the powderization stage, the metasurface was diced into COP microcubes and peeled off as the grains, each containing a single cross‐bar resonator (Figure 4d–f). The macroscopic image in Figure 4d shows numerous cubes, with the inset resolving individual meta‐grains, demonstrating the conversion from a wafer‐scale array to discrete meta‐grains. Higher‐magnification imaging in Figure 4e reveals densely packed cubes, each with a clearly defined internal cross‐bar. The oblique view in Figure 4f highlights the laminated configuration, where the metallic cross is sandwiched between two COP layers through an adhesive interlayer, forming a stable COP/adhesive/COP structure. These observations confirm that powderization yields discrete meta‐grains with preserved geometry and structural integrity, suitable for subsequent bulk reconstitution.

In the solidification stage, the meta‐grains were dispersed in a COP mixed xylene solution and cast into circular molds (Figure 4g–i). After curing and de‐molding, free‐standing bulk samples were obtained, exhibiting uniform spatial distribution and random orientation of the embedded cross‐bars within the COP matrix in (Figure 4g–h). Microscopy identified representative orientations corresponding to ${MM}_{xy}$, ${MM}_{yz}$, and ${MM}_{xz}$, confirming that isotropic dispersion of the meta‐grains can be observed in Figure 4i. The bulk structure yields an optically homogeneous composite in which lattice coupling is eliminated, whereas the intrinsic dipole resonance of each cross‐bar is preserved, forming an isotropic 3D bulk MMs for THz photonic applications.

## 4 Results and Discussion

### 4.1 Optical Characteristics of the 3D Bulk MMs

The transmission characteristics of the fabricated 3D bulk MMs were evaluated using a THz‐TDS system. A linearly polarized THz pulse was focused through a 10‐mm diameter aperture and normally incident onto the sample. The frequency range of the incident radiation extended from 0.2 to 0.5 THz with a spectral resolution of 6 GHz. The transmitted time‐domain waveform was recorded. The transmittance was defined as $T\left(f\right)=|E_{\text{sam}}\left(f\right)/E_{\text{ref}}\left(f\right){|}^{2}$, where $E_{\text{sam}}$ and $E_{\text{ref}}$ are the sample and air‐reference signals measured under identical conditions, respectively. The effective refractive index of the sample was calculated from the phase difference between the sample and the air reference, taking into account the optical path length and sample thickness. All measurements were performed with the sample surface oriented normal to the propagation direction of the THz beam.

The polarization dependence of the 3D bulk MMs was evaluated by fixing the incident polarization and then rotating the sample about the beam axis from 0° to 90°. The polarization rotation angle θ, as defined in Figure 1b, corresponds to the configuration shown in Figure 5a. Figure 5a shows the measured transmittance for different polarization rotation angles θ. All the spectra remain nearly unchanged over the entire rotation range, confirming the isotropic amplitude and low‐dispersion properties. This polarization‐independent attenuation originates from the randomly oriented meta‐grains, which support only localized dipole resonance, enabling band‐selective and omnidirectional THz attenuation. To further confirm the isotropy, oblique‐incidence measurements were also performed using a THz frequency‐domain spectrometer (THz‐FDS) equipped with a goniometer. Transmittance spectra were recorded at four incidence angles (0°, 10°, 20°, 30°) (Figure 5b). After smoothing, the spectra measured at different polarization angles and different incidence angles overlap closely, and the resonant dip remains at ∼0.36 THz.

**FIGURE 5:**
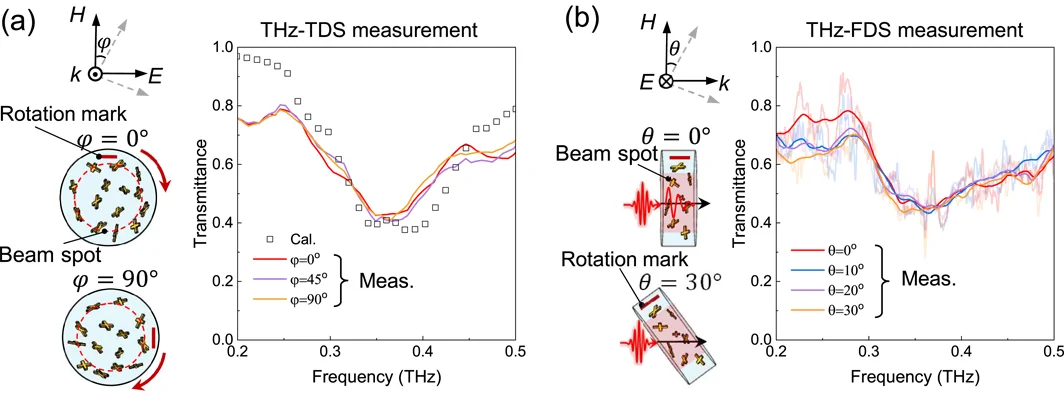
(a) Schematic illustration of the THz‐TDS measurement configuration and transmittance spectra at three polarization angles (0°, 45°, 90°) under normal incidence. (b) Schematic illustration of the THz‐FDS measurement configuration and transmittance spectra at four incidence angles (0°, 10°, 20°, 30°).

The attenuation band observed around the resonant frequency in Figure 5a is spectrally broadened compared with that of the 2D metasurface shown in Figure 3d. The experimental and simulated spectra show good agreement, confirming that the optical response of randomly oriented meta‐grains can be understood as an ensemble of incoherent resonators formed through statistical averaging. The split transmission peak observed in the numerical analysis is attributed to coherent coupling between degenerate dipolar modes with an avoided‐crossing behavior. In contrast, in the fabricated 3D bulk MMs, the amorphous distribution of cross‐bar resonators suppresses this hybridization, resulting in a broader attenuation band.

### 4.2 Application of THz Attenuators With 3D Bulk MMs

To demonstrate the tunable attenuation functionality of the 3D bulk MMs, samples with different thicknesses (t=0.507, 1.068, and 1.578 mm) were fabricated. Cross‐bar meta‐grains were uniformly dispersed at the maximum filling factor (the practical upper limit of meta‐grain loading under laboratory casting conditions) in a COP–xylene solution and cast into the mold with controlled thickness. All samples were cured under identical conditions, ensuring the same composition while varying only the total thickness, as shown in Figure 6a. Optical microscope insets reveal that the t=0.507 mm sample consists of a single layer of meta‐grains, corresponding to a near‐planar configuration analogous to a 2D metasurface but with de‐latticed, randomly oriented resonators. In contrast, the t=1.068 and 1.578 mm samples contain overlapping layers of meta‐grains along the light‐propagation directions.

**FIGURE 6:**
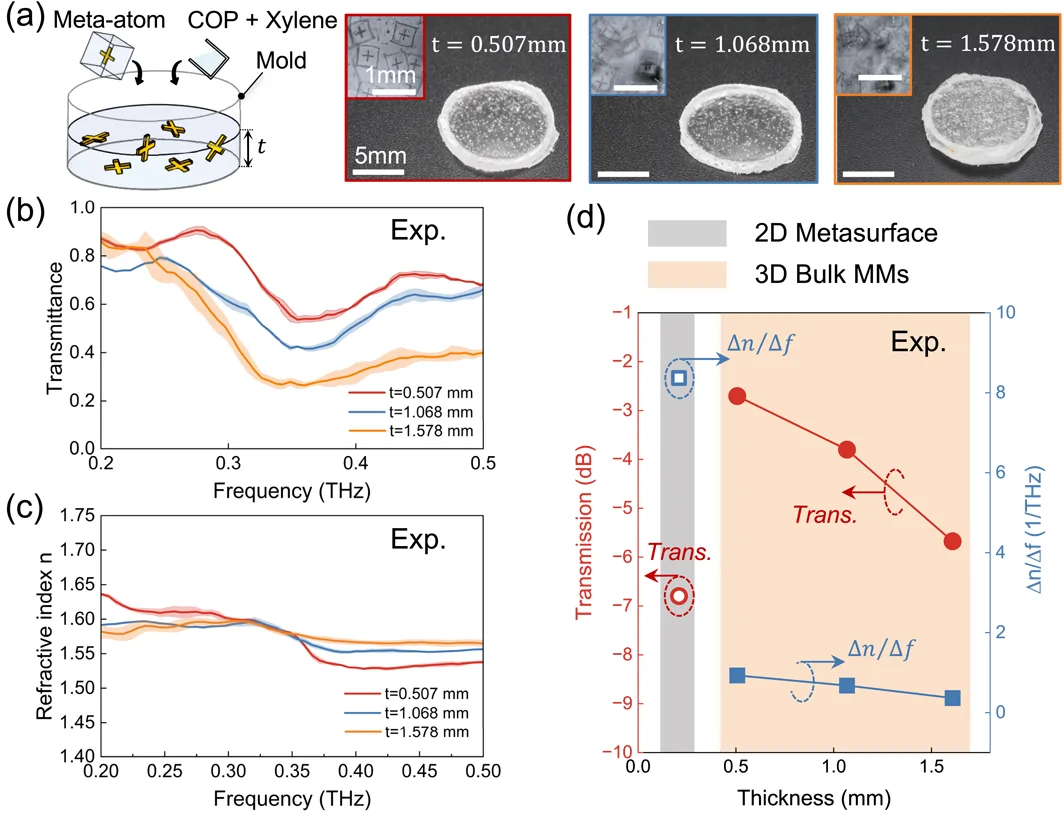
(a) Schematic illustration (left) and optical microscope images (right) of 3D bulk MMs samples with different thicknesses. (b, c) Measured transmittance and corresponding refractive‐index spectra for the three samples. The shaded bands indicate the variation range over polarization rotation angles α=0°–90°. (d) Summary of transmittance (red circles) and dispersion slope Δn/Δf (blue squares) as functions of sample thickness, together with the 2D metasurface optical properties (red circle ring for transmittance and blue square ring for the dispersion slope).

These thickness‐controlled samples enable a direct evaluation of THz transmission as a function of propagation distance within the effective medium of the 3D bulk MMs. The measured THz transmittance and refractive‐index *n* for these three samples are presented in Figure 6b,c, respectively.

For all sample thicknesses, a clear dipole resonance peak appears near 0.36 THz, confirming that the fundamental electromagnetic response of cross‐bar resonators is preserved even when randomly dispersed within the bulk medium. This resonance originates from dipolar surface currents excited on the metallic cross‐bars, which re‐radiate and interfere with the transmitted field. The robustness of the resonance peak across all sample thicknesses indicates that the local scattering behavior of each cross‐bar resonator is independent of the global configuration. As the thickness increases, the attenuation of the resonance peak becomes stronger, as described by Equation (5). This tunable attenuation originates from the incoherent nature of the resonators, in which thicker bulk MMs provide a longer optical path for multiple dipolar excitations along the propagation direction. Because the meta‐grains are encapsulated in COP and randomly oriented, coupling between neighboring dipoles occurs without fixed phase relations, suppressing coherent interference and preventing mode splitting or spectral distortion. Owing to the de‐latticing strategy, the refractive‐index spectra near the dipole resonance exhibit low dispersion regardless of thickness, with Δn≈0.053 from 0.34 to 0.4 THz for the t=0.507 mm sample (Figure 6c). As the propagation path increases, the phase fluctuations induced by individual incoherent resonators are statistically averaged out, resulting in smoother effective phase accumulation and a nearly dispersion‐free behavior in thicker samples.

The dependence of attenuation and dispersion slope Δn/Δf on the sample thickness is summarized in Figure 6d, together with the 2D metasurface shown in Figure 3d,e. As the thickness increases, the transmittance gradually decreases across the dipole resonance, indicating that the attenuation level can be continuously tuned by controlling the propagation length within the same composite medium. This tunability is achieved while maintaining optical isotropy, enabling a metasurface equivalent attenuation function in a fully de‐latticed 3D form. Meanwhile, the spectral slope of the refractive index Δn/Δf becomes progressively flatter as phase incoherence among scattered waves increases, resulting in an almost dispersion‐free response. In addition to the sample thickness, the optical response can also be tuned by changing the meta‐grain filling factor during fabrication. As shown in Supporting Information S1: Figure S4, increasing the filling factor at a fixed thickness (t=0.51mm) deepens the attenuation without shifting the resonant frequency, whereas the refractive‐index dispersion remains low.

Compared with the 2D metasurface, which exhibits a dispersion slope of Δn/Δf≈8.37, the 3D bulk MMs show a much smaller value of ∼0.93 for the t=0.507 mm sample, and as low as ∼0.36 for t=1.578 mm. This represents an order‐of‐magnitude suppression of dispersion, confirming that coherent coupling between neighboring dipoles is effectively removed, whereas the intrinsic dipole resonance remains stable without spectral distortion. The 3D bulk MMs thus provide a robust and structurally scalable platform for band‐selective, low‐dispersion, and thickness‐tunable THz attenuation, offering a simple route to THz system integration without additional phase‐compensation components.

### 4.3 Discussion

The 3D bulk MMs presented in this study exhibit a band‐selective, isotropic, and thickness‐tunable THz response with strongly suppressed dispersion at the resonant frequency. As the sample thickness increases from 0.507 to 1.578 mm, the transmittance decreases continuously within the same composite medium, whereas the dispersion slope Δn/Δf is reduced from 8.37 for the 2D metasurface to 0.36 for the thickest 3D bulk sample. This order‐of‐magnitude reduction indicates that the de‐latticing strategy removes the lattice‐coupled phase response of the periodic metasurface while preserving the localized dipole resonance of the cross‐bar. As a result, the bulk response remains band‐selective, but the refractive‐index variation becomes much smoother around the attenuation band. The macroscopic optical response is reproducible across the independently fabricated samples of different thicknesses and filling factors reported above, confirming that the random dispersion is controlled and reproducible at the level that governs the optical response.

De‐latticing reduces the attenuation depth relative to the original 2D metasurface. Because the meta‐grains are randomly oriented, only a fraction of the embedded cross‐bars are optimally aligned with the incident electric field at any given time, so the effective resonant filling factor is lower and the attenuation dip per unit thickness is shallower. The attenuation depth can be recovered by increasing the sample thickness or the filling factor, as shown in Figure 6d and Supporting Information S1: Figure S4, without compromising the low‐dispersion character of the bulk response. Random orientation reduces the resonant coupling of each grain by a fixed orientation‐averaging factor, not by an open‐ended trade‐off. The isotropy is set by the random orientation, whereas the attenuation depth is set independently by the thickness and filling factor, so this fixed reduction is compensated and the response stays isotropic as the attenuation increases.

The remaining disagreement between the experiment and simulation is confined to the band edges and does not affect the interpretation of the attenuation mechanism. Below ∼0.25 THz, the deviation is minor, whereas the additional transmission decrease above ∼0.45 THz is attributed to scattering from micro‐bubbles in the thick bulk samples. The numerical model is built from 2D unit‐cell simulations projected and averaged over orientation, so it does not capture the full 3D multiple scattering among the randomly arranged grains in a thick sample, which is the origin of the residual band‐edge deviation for the bulk. Importantly, the resonant attenuation near 0.36 THz remains governed by the metallic cross‐bar resonators, as confirmed by the pure COP control measurement (see Supporting Information S1: Figure S5), which shows no attenuation feature across the full band. The high‐frequency reduction is therefore a fabrication‐related effect outside the main attenuation band, and vacuum processing would be important in the practical fabrication of thicker devices to maintain high transparency outside the target frequency band. Alternatively, replacing the metallic resonators with low‐loss dielectric meta‐atoms could further reduce off‐resonant loss while preserving tunable attenuation characteristics [51].

Table 1 compares the proposed 3D bulk MMs with three representative THz attenuator architectures: stacked metal‐insulator attenuators [46], stacked SRR films on flexible substrates [47], and multilayer graphene attenuators [48]. “Disp.,” “Angle dep.,” and “Fab. scalability” denote dispersion, angle dependence, and fabrication scalability, respectively. Unlike the layered 2D structures in the table, the present bulk medium is three‐dimensionally isotropic, shows low dispersion, and allows the attenuation level to be adjusted through the filling factor and sample thickness.

**TABLE 1:** Comparison with representative THz attenuators.

Attenuator type	Bandwidth	Disp.	Angle dep.	Fab. scalability	Features
3D bulk MMs (this work)	∼33% FWHM	Low	Low	Molding/casting	Thickness‐tunable
Stacked metal‐insulator	∼48% FWHM	High	High	Photolithography	Layered structure
Stacked SRR films	4.2× single layer	Moderate	High	Femtosecond laser lithography	Layered structure
Multilayer graphene	Broadband	High	Low	CVD + stacking	Electrical tunability

## 5 Conclusion

In summary, we have proposed and experimentally demonstrated 3D bulk MMs that exhibit optical isotropy in THz attenuation, based on a de‐latticed cross‐bar resonator strategy. By powderization of a periodic cross‐bar metasurface into randomly dispersed meta‐grains embedded within a COP matrix, the intrinsic dipole resonance of each meta‐atom is preserved, whereas long‐range lattice coupling and coherent interference are eliminated. The fabricated structures exhibit controllable transmission governed by the propagation length and meta‐grain filling factor, enabling thickness‐tunable attenuation with a low‐dispersion refractive index. This alignment‐free and scalable process demonstrates an isotropic, low‐dispersion 3D bulk MMs attenuator that bridges planar metasurface design with volumetric THz photonic functionality, addressing a capability that polarization‐ and angle‐dependent 2D attenuators cannot reach. Such structures provide a practical platform for THz power regulation, band‐selective spectral control, and signal conditioning, and the underlying concept can be extended to other frequency ranges and material systems for dispersion‐engineered wave manipulation in bulk photonic media.

## Author Contributions

**Zhen Liu:** methodology, conceptualization, writing – original draft, writing – review and editing. **Chikaho Nagashima:** validation, investigation, writing – original draft. **Yoshiaki Kanamori:** conceptualization, writing – review and editing, funding acquisition, methodology, supervision.

## Funding

This work was supported by the JST Program for co‐creating startup ecosystem (Grant JPMJSF2312). This work was supported by Micro/Nano‐Machining Research and Education Center, Tohoku University.

## Conflicts of Interest

The authors declare no conflicts of interest.

## Supporting information


Supporting Information S1

## Data Availability

The data that support the findings of this study are available from the corresponding author upon reasonable request.
