# Supplementary material for: Band‐Selective, Low‐Dispersion Terahertz Attenuators Based on 3D Bulk Metamaterials Leveraging De‐latticed Cross‐Bar Resonator Ensembles
Source: Nanophotonics. 2026 Aug 13;15(15):e70203. doi: 10.1002/nap2.70203 (PMC13642165; doi:10.1002/nap2.70203)
Supplement: Supplementary file 1 [file NAP2-15-e70203-s001.pdf]

Supplementary Material

Band-Selective, Low-Dispersion Terahertz Attenuators Based on 3D Bulk Metamaterials Leveraging De-latticed Cross-bar Resonator Ensembles

Zhen Liu[1], Chikaho Nagashima[1], and Yoshiaki Kanamori[1]

[1]Graduate School of Engineering, Tohoku University,
Aoba 6-6-01 Aramaki, Aoba-ku, Sendai 980-8579, Japan

S1. Response of a single meta-grain

Numerical simulations were performed for an isolated meta-grain using the Time Domain Solver in CST Microwave Studio. Periodic lattice coupling was removed by setting all six boundaries to “Open (add space),” so that the response of a single grain could be evaluated directly. The broadband radar cross-section (RCS) spectra in Fig. S1(a) show that the 0.2–0.5 THz operating window contains a single isolated resonance, and that the next higher-order radiative resonances do not appear until above 0.6 THz.

The field distributions at resonance [Fig. S1(c)] are consistent with a fundamental electric dipole mode. The surface current flows linearly along the arm parallel to the incident electric field, the electric field concentrates at the two arm ends, and no circulating loop current is observed. Within the simulated band, no clear magnetic resonance is observed, consistent with the absence of a loop-current path in the cross-bar geometry. We also do not observe signatures of trapped or dark modes, as expected for the fully symmetric structure under the present excitation. The 0.2–0.5 THz response is therefore dominated by a single electric dipole resonance.

This single dipole resonance is described by a Lorentzian polarizability,

$$\alpha(f) = \frac{A}{f_0^2 - f^2 - i\gamma f}, \tag{S1}$$

where f is the frequency, f_0 the resonance frequency, γ the damping rate that sets the resonance linewidth, and A a constant amplitude. Since the scattered power obeys RCS $\propto |\alpha(f)|^2$, fitting the RCS spectra of Fig. S1 to this form gives f_0 and γ, and hence the quality factor $Q = f_0/\gamma$. The fitted resonance frequency is 0.37, 0.34, and 0.32 THz for L = 230, 260, and 290 μm, following the half-wave dipole model of Eq. (1) of the main text. For $L = 260$ μm the resonance at 0.34 THz coincides with the measured attenuation dip of the bulk near 0.36 THz, which identifies the bulk dip as the cross-bar dipole resonance. In every case the linewidth is broad, with $Q \approx 6$; this broad single-grain response is the quantity carried into Sections S2 and S3.

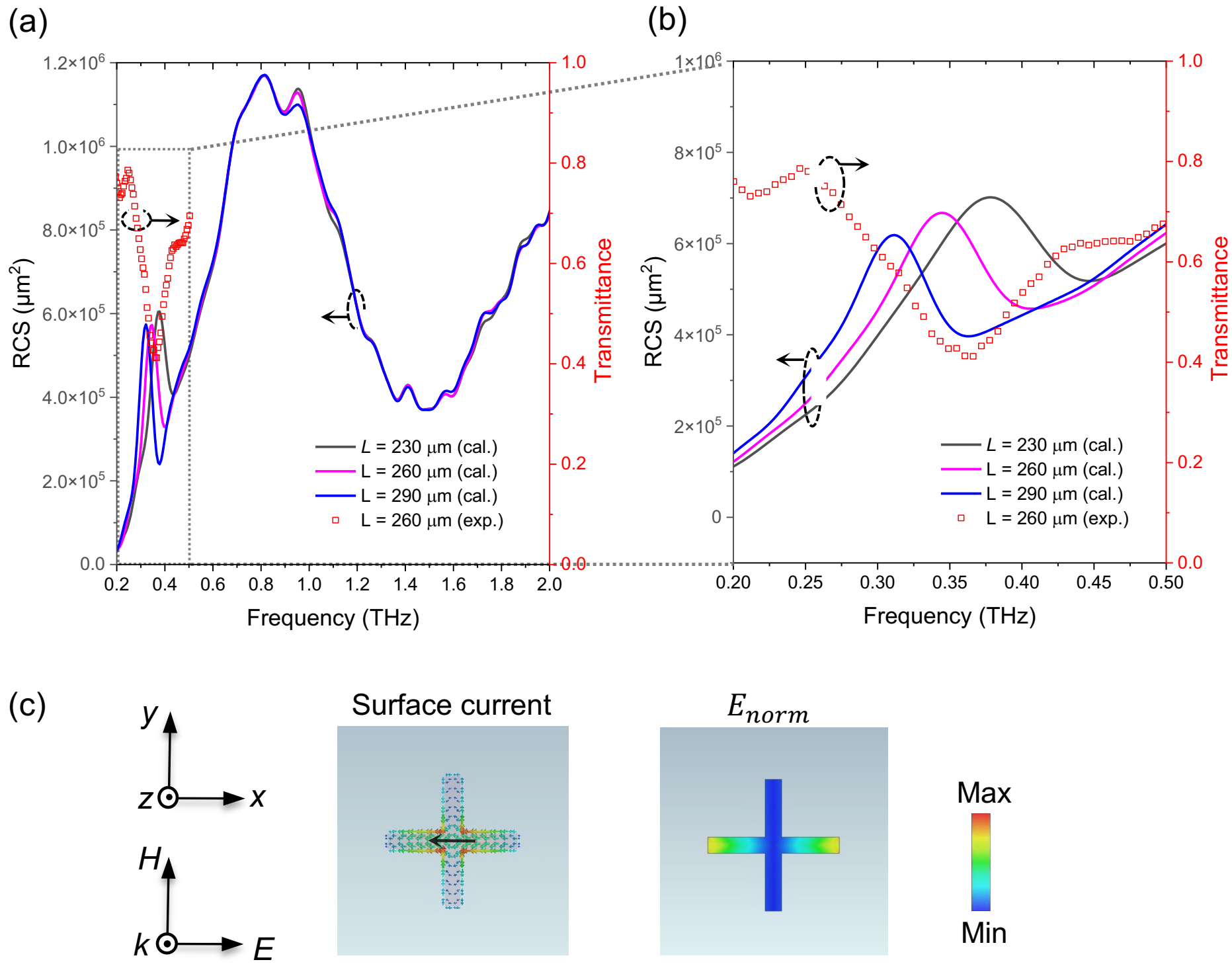

Figure S1. (a) Broadband radar cross-section (RCS) spectra of isolated meta-grains with arm lengths $L = 230$, 260, and 290 μm, together with the measured transmittance of the bulk sample for $L = 260$ μm. (b) Zoomed view of the 0.2–0.5 THz operating window, showing a single isolated resonance whose frequency shifts with arm length and agrees with the measured attenuation dip. (c) Simulated surface-current and electric-field distributions at resonance, confirming a fundamental electric dipole mode of the cross-bar.

S2. Orientational averaging of the meta-grains

In the bulk the grains are randomly oriented, so the macroscopic response can be approximated by averaging the single-grain response of Section S1 over all orientations. We collect the single-grain responses along the three Cartesian axes into a polarizability tensor $\bar{\bar{\alpha}}$, whose diagonal components α_{xx}, α_{yy}, and α_{zz} are represented by the three orientations MM_{xy}, MM_{yz}, and MM_{xz}. For a statistically isotropic ensemble, orientational averaging gives the scalar effective polarizability as one third of the tensor trace [1, 2],

$$\langle \alpha \rangle = \frac{1}{3}\,\mathrm{Tr}(\bar{\bar{\alpha}}) = \frac{1}{3}\big(\alpha_{xx} + \alpha_{yy} + \alpha_{zz}\big), \tag{S2}$$

where $\langle \alpha \rangle$ is the orientation-averaged polarizability and $\mathrm{Tr}(\bar{\bar{\alpha}})$ is the trace of the polarizability tensor. This relation is exact for the orientational average of a second-rank polarizability tensor under a fully random orientation distribution. In the present model, it provides the basis for constructing a discrete three-orientation approximation.

The weighting of the face-on orientation can also be understood from the field projection onto the cross-bar plane. The cross-bar lies in a plane and is driven only by the component of the incident electric field that lies within that plane; the perpendicular component does not drive current along the arms. Let E be the amplitude of the incident electric field and let θ be the angle between the incident electric field and the normal to the cross-bar plane. The in-plane

field amplitude is then

$$E_{\parallel} = E \sin\theta, \tag{S3}$$

where $E_{\parallel}$ is the component of the incident field parallel to the cross-bar plane. Since the coupling strength scales with the field intensity, the normalized in-plane coupling factor is

$$\frac{|E_{\parallel}|^2}{|E|^2} = \sin^2\theta. \tag{S4}$$

Averaging this factor over a uniform distribution of plane normals gives

$$\left\langle \sin^2\theta \right\rangle = \frac{1}{4\pi}\int_0^{2\pi}\int_0^{\pi} \sin^2\theta \, \sin\theta \, d\theta \, d\phi = \frac{2}{3}, \tag{S5}$$

where ϕ is the azimuthal angle and the factor $\sin\theta \, d\theta \, d\phi$ is the solid-angle element on the unit sphere. Thus, a randomly oriented cross-bar couples, on average, with two thirds of the strength of an aligned face-on resonator.

This continuous result provides a useful constraint on the discrete orientational model used in the main text. As shown in Fig. S2, the discrete rotations by 0°, 90°, 180°, and 270° about the x, y, and z axes generate 12 configurations. Owing to the C_{4v} symmetry of the cross-bar, these configurations reduce to three electromagnetically inequivalent orientations: MM_{xy}, MM_{yz}, and MM_{xz}. Within this discrete representation, 8 of the 12 configurations are equivalent to MM_{xy}, while the remaining 4 are divided equally between MM_{yz} and MM_{xz}, giving

$$\beta_{xy} = \frac{8}{12} = \frac{2}{3}, \quad \beta_{yz} = \frac{2}{12} = \frac{1}{6}, \quad \beta_{xz} = \frac{2}{12} = \frac{1}{6}. \tag{S6}$$

The resulting face-on weight, $\beta_{xy} = 2/3$, matches the continuous orientational average of the in-plane coupling factor in Eq. (S5). We therefore use this discrete set as a symmetry-based approximation to the random ensemble, constrained to reproduce the mean coupling strength of a fully random orientation distribution.

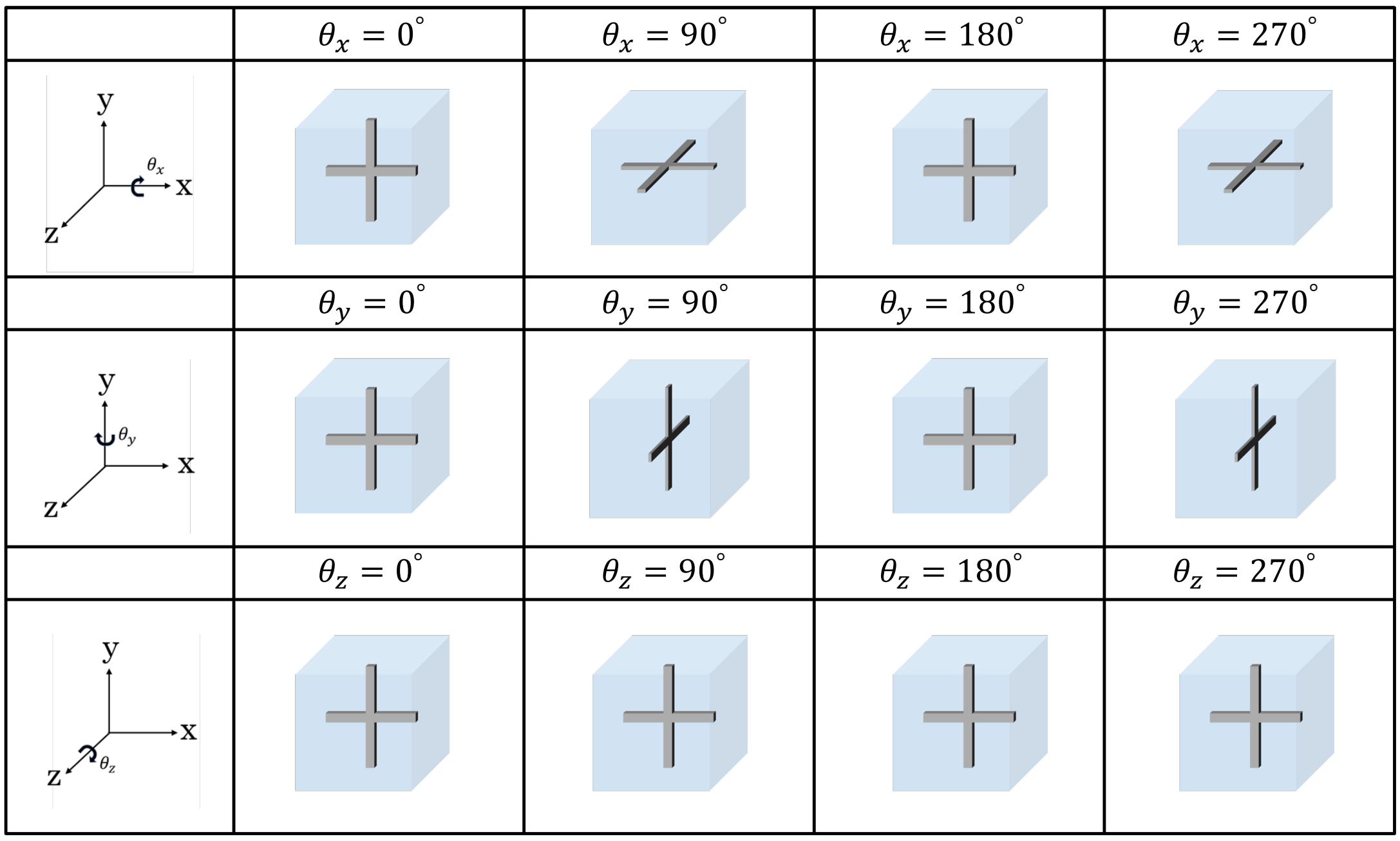

Figure S2. Orientations of a cubic meta-grain under discrete rotations about the x, y, and z axes. Owing to the C_{4v} symmetry of the cross-bar, the 12 configurations reduce to three electromagnetically inequivalent orientations: MM_{xy}, MM_{yz}, and MM_{xz}. These orientations form the symmetry-based discrete representation used for orientational averaging.

The transmittance is a transmission quantity and is therefore obtained from the S-parameters of the metasurface unit cell in each orientation, rather than from the isolated-grain RCS of Section S1. For each orientation i, the transmittance is first averaged over the incidence angle ϕ,

$$\langle T_i(f)\rangle_\phi = \left\langle\, |S_{21,i}(\phi, f)|^2 \,\right\rangle_\phi, \tag{S7}$$

where $S_{21,i}(\phi, f)$ is the complex transmission coefficient of orientation i at incidence angle ϕ and frequency f. The three orientations are then combined with the weights of Eq. (S6) to give the single-layer bulk transmittance,

$$T_{\text{bulk}}(f) = \sum_i \beta_i \,\langle T_i(f)\rangle_\phi, \tag{S8}$$

where β_i is the orientation weight of orientation i. For a sample comprising $N \approx t/p$ meta-grain layers, where t is the sample thickness and p is the layer spacing, assuming incoherent stacking and neglecting coherent Fabry–Pérot interference between layers gives the total transmittance

$$T^{(N)}_{\text{bulk}}(f) = \left[\, T_{\text{bulk}}(f) \,\right]^N. \tag{S9}$$

These three relations are Eqs. (3)–(5) of the main text.

S3. Analysis of the low-dispersion mechanism

Sections S1 and S2 explain the isotropic transmittance of the bulk. The reduced dispersion, however, is not a consequence of orientational averaging alone. Instead, it results from the de-latticing strategy, which removes the periodic lattice coupling responsible for the sharp dispersive response of the 2D metasurface. We analyze this point below by comparing the isolated-grain response with the periodic metasurface response.

In the periodic 2D metasurface each cross-bar is driven by the incident field and by the fields scattered from all the others. Within the coupled-dipole description of periodic arrays [3], the effective polarizability of a cross-bar in the lattice is

$$\alpha_{\text{eff}}(f) = \frac{1}{\alpha(f)^{-1} - C(f)}, \tag{S10}$$

where $\alpha(f)$ is the single-grain polarizability of Section S1 and $C(f)$ is the lattice sum of the fields arriving at one cross-bar from all the others. Near the in-plane diffraction condition of Eq. (2) of the main text, $C(f)$ varies steeply and approaches $\alpha(f)^{-1}$, so $\alpha_{\text{eff}}(f)$ becomes large and sharp. This surface-lattice resonance (SLR), hybridized with the dipole resonance, produces the steep, angle-dependent index $n_{\text{eff}}(\phi, f)$ of Figs. 2(a) and 2(b) and the large slope $\Delta n/\Delta f \approx 8.37$. The lattice thus turns the broad single-grain dipole of Section S1 ($Q \approx 6$) into a narrow collective resonance, and this sharpening is what makes the dispersion of the 2D metasurface large.

We confirmed this by comparing the transmission phase of the periodic 2D metasurface with that of an isolated meta-grain, calculated under periodic and open boundary conditions respectively. Figure S3(a) shows that the periodic metasurface exhibits a sharp phase jump of approximately 4 rad near 0.35 THz, whereas the isolated meta-grain shows a smooth, nearly linear phase variation across the 0.2–0.5 THz band. The phase-slope term in Fig. S3(b) reaches a peak of approximately +650 rad/THz at resonance for the metasurface but remains below 50 rad/THz for the isolated grain, a reduction of more than one order of magnitude. The low isolated-grain slope is the direct signature of the broad single-grain resonance of Section S1 ($Q \approx 6$), in contrast to the sharp 2D SLR. Once the long-range periodicity is removed, the lattice sum

no longer contains the sharp diffractive feature required to form the SLR, and the response is therefore governed primarily by the broad single-grain dipole resonance.

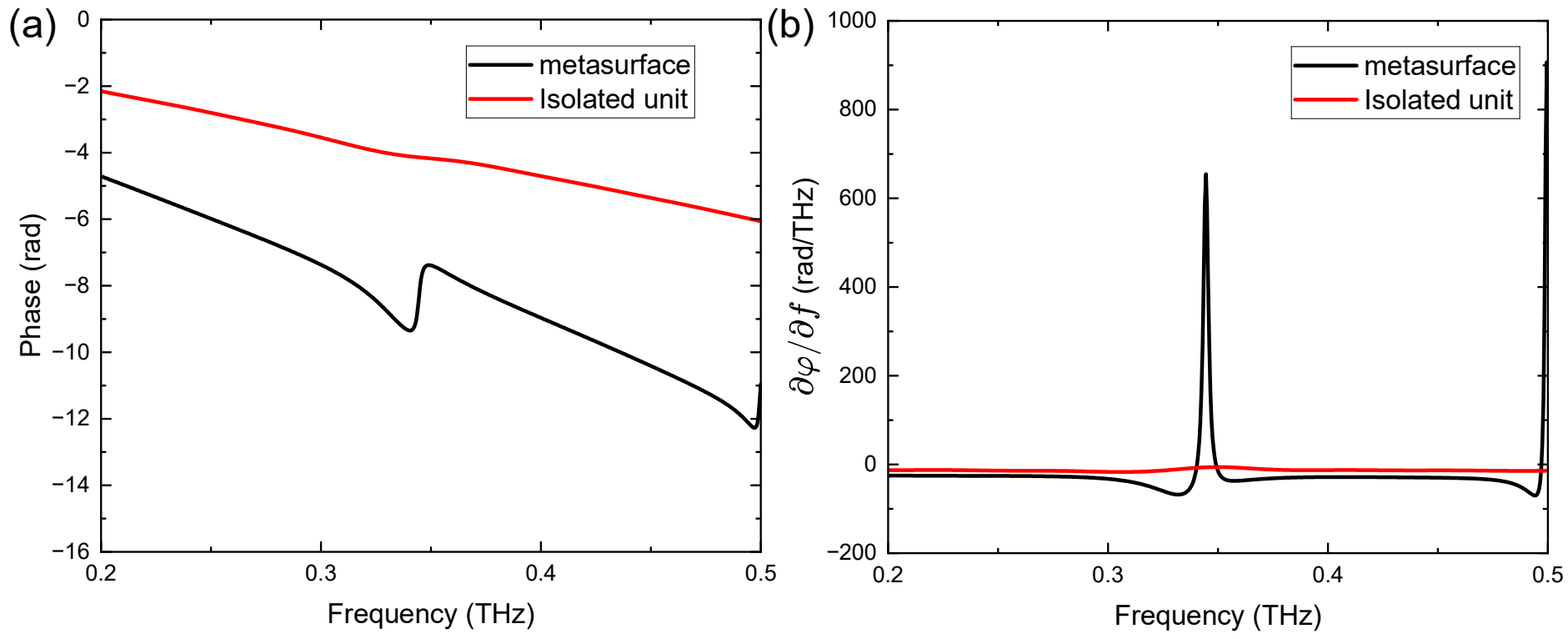

Figure S3. (a) Transmission phase of the periodic 2D metasurface and the isolated meta-grain. (b) Corresponding phase-slope term $\partial\varphi/\partial f$.

Consistent with this picture, the dispersion slope measured by THz-TDS falls from $\Delta n/\Delta f \approx 8.37$ for the 2D metasurface to 0.36 for the thickest bulk sample, an order-of-magnitude reduction obtained directly from the data.

Finally, we state the regime in which the bulk is described, since the grain size is not far below the wavelength. After curing, the polymer grains merge with the surrounding matrix, so the cured composite retains no periodic length scale, and the only remaining feature is the metallic cross-bar, about 260 μm in size. At the resonance the wavelength in the COP is about 560 μm, so the cross-bar is comparable to it, roughly half a wavelength. The composite therefore lies at the subwavelength boundary rather than deeply within the quasi-static limit, and we do not treat it as a strict effective medium. Consistent with this, the numerical model evaluates an orientation-averaged transmittance [Eqs. (S7)–(S9)] and extracts no refractive index, while the effective index reported in the main text is obtained directly from the measured macroscopic transmitted phase of the millimetre-thick samples. The broad, low-dispersion response of the bulk is thus carried over from the single broad dipole of Section S1 once the lattice coherence is removed, and it does not rely on a quasi-static homogenization.

S4. Effect of meta-grain filling factor on optical properties

Samples with meta-grain filling factors of 25%, 50%, and 100% were prepared at a fixed thickness of $t = 0.51$ mm. As the filling factor increases, the attenuation deepens while the resonant frequency shifts little [Fig. S4(a)]: the transmittance minimum drops from 0.72 at 25% to 0.64 at 50% and 0.54 at 100%. The resonant frequency is set by the cross-bar geometry and is insensitive to the number density of meta-grains.

The effective refractive index rises with filling factor, consistent with effective-medium behavior, and the spectra remain smooth across the resonance band [Fig. S4(b)]. The linear fits in Fig. S4(c) show that the attenuation depth scales with filling factor, while the dispersion metric stays small across the full range. The filling factor therefore controls attenuation and refractive index independently of the resonance frequency, without degrading the low-dispersion character of the 3D bulk MMs.

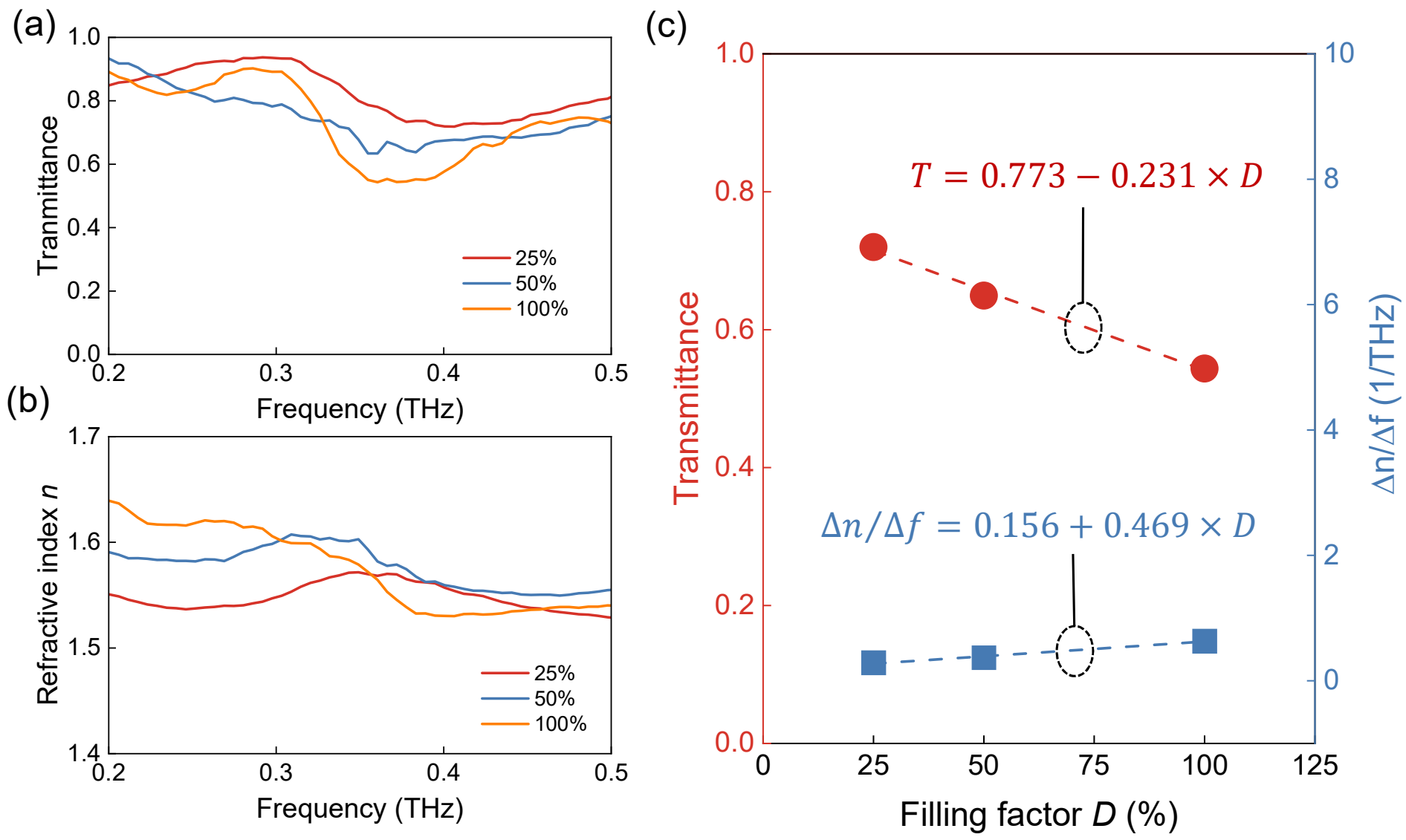

Figure S4. Optical response of samples with meta-grain filling factors of 25%, 50%, and 100% at a fixed thickness of $t = 0.51$ mm. (a) Transmittance spectra. (b) Effective refractive index. (c) Linear relations of the minimum transmittance and the dispersion metric with filling factor. Increasing the filling factor deepens the attenuation and raises the effective refractive index, while the dispersion remains low.

S5. Thick pure COP bulk

A pure COP bulk sample with a thickness of $t = 1.51$ mm was prepared by hot pressing and measured in the same frequency range. As shown in Fig. S5, the sample remains highly transparent across the measured band and shows no resonant dip near 0.35 THz, confirming that the attenuation dip observed in the 3D bulk MMs originates from the metallic cross-bar resonators rather than the COP matrix. The weak oscillatory modulation in the spectrum is attributed to Fabry–Pérot interference within the finite-thickness slab.

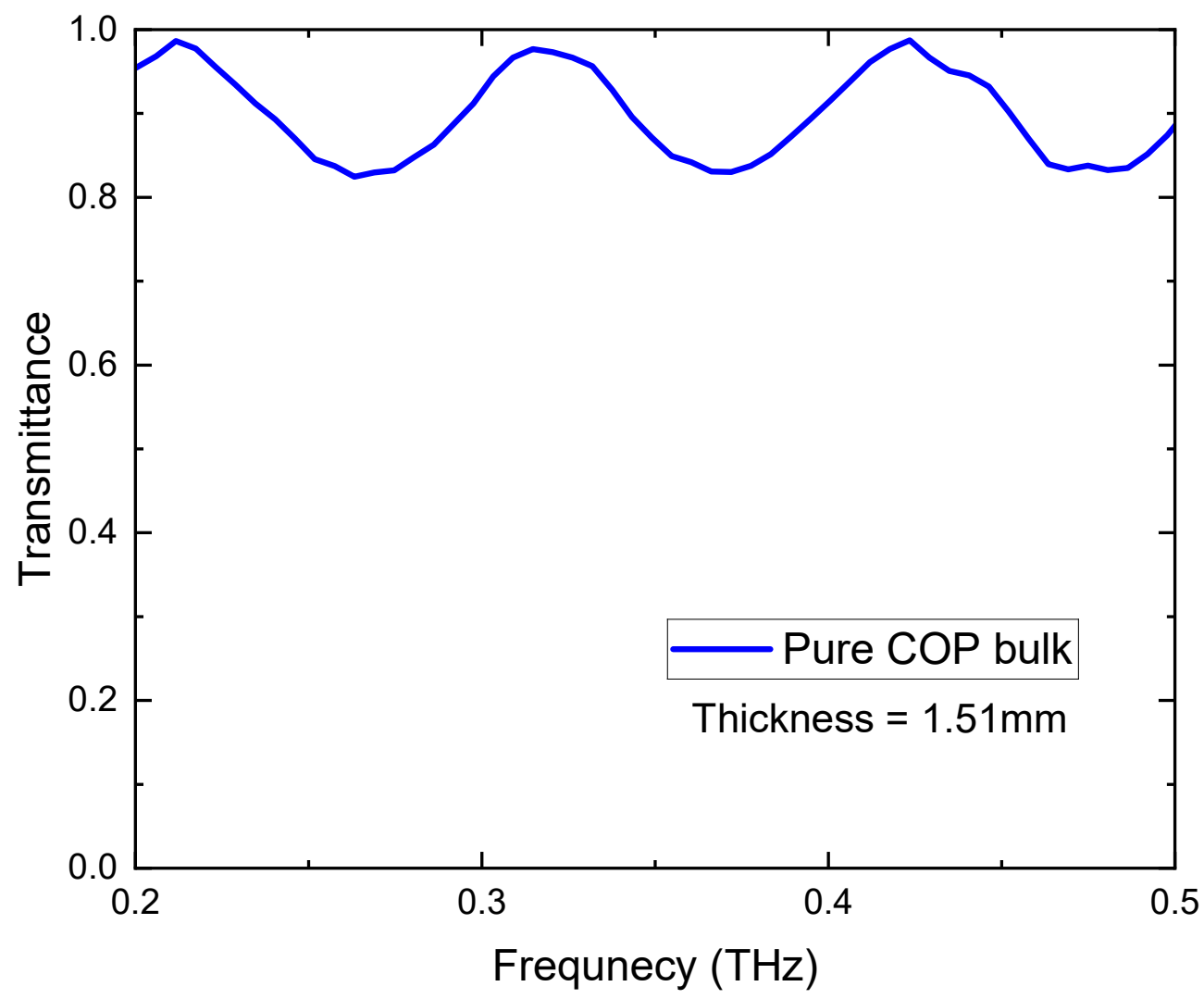

Figure S5. Transmittance spectrum of a pure COP bulk sample with $t = 1.51$ mm prepared by hot pressing.

References

[1] C. F. Bohren, D. R. Huffman, *Absorption and Scattering of Light by Small Particles*, Wiley-VCH, Weinheim, Germany, **1998**.

[2] A. Sihvola, *Electromagnetic Mixing Formulas and Applications*, Number 47 in IEE Electromagnetic Waves Series. Institution of Engineering and Technology, London, **1999**.

[3] F. J. García de Abajo, *Reviews of Modern Physics* **2007**, *79*, 4 1267.
